# Videoconferencing Psychotherapy in the Public Sector: Synthesis and Model for Implementation

**DOI:** 10.2196/14996

**Published:** 2020-01-21

**Authors:** Samuel David Muir, Kathleen de Boer, Neil Thomas, Elizabeth Seabrook, Maja Nedeljkovic, Denny Meyer

**Affiliations:** 1 Centre for Mental Health Swinburne University of Technology Hawthorn Australia

**Keywords:** implementation science, videoconferencing, psychotherapy, public sector, telemedicine, mental health

## Abstract

**Background:**

Videoconferencing psychotherapy (VCP) is a growing practice among mental health professionals. Early adopters have predominantly been in private practice settings, and more recent adoption has occurred in larger organizations, such as the military. The implementation of VCP into larger health service providers in the public sector is an important step in reaching and helping vulnerable and at-risk individuals; however, several additional implementation challenges exist for public sector organizations.

**Objective:**

The aim of this study was to offer an implementation model for effectively introducing VCP into public sector organizations. This model will also provide practical guidelines for planning and executing an embedded service trial to assess the effectiveness of the VCP modality once implemented.

**Methods:**

An iterative search strategy was employed, drawing on multiple fields of research across mental health, information technology, and organizational psychology. Previous VCP implementation papers were considered in detail to provide a synthesis of the barriers, facilitators, and lessons learned from the implementation attempts in the military and other public sector settings.

**Results:**

A model was formulated, which draws on change management for technology integration and considers the specific needs for VCP integration in larger organizations. A total of 6 phases were formulated and were further broken down into practical and measurable steps. The model explicitly considers the barriers often encountered in large organizational settings and suggests steps to increase facilitating factors.

**Conclusions:**

Although the model proposed is time and resource intensive, it draws on a comprehensive understanding of larger organizational needs and the unique challenge that the introduction of VCP presents to such organizations.

## Introduction

### Background

The provision of health care services on the Web via real-time video communication is growing [1,2]. In the delivery of mental health care services, particular growth has been seen in the form of videoconferencing psychotherapy (VCP) [3]. VCP is appealing because it emulates the face-to-face delivery of traditional in-person treatment, while offering a number of potential advantages, including increased flexibility and reach. Moreover, VCP has the potential to overcome challenges, such as time constraints, scheduling difficulties, and client concerns about treatment-seeking stigma, by allowing clients to engage with professional services in the privacy of their own home [4-7]. Arguably the greatest advantage of VCP is its ability to overcome access-to-care barriers for those in underserviced regions. This advantage makes VCP an attractive service offering for organizations that serve geographically disperse or isolated populations, such as military personnel, veterans, prison inmates and staff, first responders, mining workers, or people living in rural or remote locations [8-10].

Although a growing body of research has found VCP to be effective [11], studies have also reported difficulties implementing the modality, with VCP researchers and experts expressing concerns at how VCP initiatives often fail to progress past the pilot phase to implementation on a wider scale [12-14]. This is partly because of the nature of the organizations themselves—large geographically dispersed health care organizations where the complexity of implementation is often underestimated [14,15].

It appears that introducing VCP services within organizations already providing services can be quite disrupting as implementation and governance of VCP requires input from a wide variety of parties (eg, the health care providers, funding bodies, and software providers) and personnel (eg, clinical staff, information technology [IT] staff, management, and policy makers). Therefore, VCP implementation in public sector settings represents a significant multifaceted challenge, requiring various changes and collaborations that have implications at an individual and organizational level. A potential explanation for the lack of successful widespread implementation in these settings may be because of a lack of clarity around how to best implement and sustain VCP modalities in real-world settings. Although considerable research has been conducted, examining the effectiveness of VCP [11] and developing best practice guidelines [16-18], there does not appear to be a clear consensus as to how best to implement such services. This has led to researchers calling for rigorous implementation models to be developed and tested [14,19-21].

### This Study

This paper aimed to synthesize the findings and lessons learned from previous implementation attempts into a cohesive and integrated model, specifically for the implementation of VCP in public sector organizational settings. In addition to previously developed models and best practice guidelines, the model proposed here draws on the barriers and facilitators for the implementation and delivery of VCP identified in earlier papers, as well as the solutions developed.

## Methods

To develop a comprehensive and cohesive model for VCP implementation, this study utilized an iterative search strategy to bring together insights from mental health, IT, and organizational psychology. As such, a broad range of search terms, including (but not limited to) variations of implementation, videoconference, psychotherapy, mental health, and telehealth, were entered into searches across PubMed, PsycINFO, and Web of Science databases. This study focused on synthesizing the barriers and facilitators of VCP implementation, identified in previous studies and the previous models presented for VCP implementation in large or public sector organizations. Our model also incorporates learning from other fields of research (eg, IT and organizational psychology) in the emergent recommendations.

## Results

### Brief Review of Barriers and Facilitators of Videoconferencing Psychotherapy Implementation

A review of the VCP literature revealed that there are several barriers to implementing this modality in public sector organizations. Common barriers to VCP implementation efforts include clinician attitudes and availability, as well as technological and logistical issues [12,22-24]. Additional barriers for public sector organizations implementing VCP services include concerns regarding clinical risk management and data security, resource and funding constraints, regimented protocols, and geographically dispersed service providers [4]. For example, Brooks et al [25] surveyed 39 stakeholders who were involved in the implementation of VCP services for veterans in the United States. Respondents noted many challenges with the implementation process, including staffing issues, setting up the VCP infrastructure, obtaining trust and acceptance of the new technology from staff and clients, and recruitment. Similarly, Adler et al [26] conducted a pilot study, investigating VCP implementation for the US Department of Veterans’ Affairs (VA). Interviews with clinicians involved in the study found that all 6 sites suffered delays to implementation because of unanticipated organizational constraints (eg, limited space, misplaced equipment, and difficulties setting up and supporting new technology). Further barriers included inadequate staffing, delays in staff training and poor communication with clinical personnel regarding priorities and workload, as well as issues with staff changes.

Although much of the research investigating VCP implementation has originated in the US VA [12,22,25-31], there are several further organizational contexts in the public sector where VCP has been implemented, such as schools, hospital emergency departments, and palliative care settings [32-34]. For example, Donley et al [33] investigated VCP implementation in an emergency department. The authors highlighted several factors that are important to consider in terms of implementing VCP in this setting, such as reviewing the technology and resources required and staffing considerations. Furthermore, the authors highlighted that, as the use of VCP is a relatively new process in the ED, guidelines and policies will need to be developed. The authors also noted the importance of ensuring that the development of workflow processes is contributed to by all the teams involved in VCP and that this should involve clear communication regarding roles and responsibilities.

### Brief Review of Previous Implementation Models

Shore and Manson [10] provided an early attempt at prescribing a model for VCP implementation in rural US settings for veteran American Indians. This model included 6 stages: (1) establishing the willingness of the target population to engage with the modality, (2) a survey of resources required for implementation, (3) consideration of the involvement of external parties, (4) drafting protocols and assigning roles, (5) an initial pilot study, and (6) complete integration within the organization. This model was found to be successful in implementing and sustaining VCP in the US VA [10]. Shore and Manson [10] highlighted that the timeline for implementation at each site was largely dependent on the participation and buy-in from staff within the various sites. In their case, stages 1 to 4 took 12 months, whereas the pilot study (stage 5) lasted 6 months. This was the only previous model specific to VCP implementation found in our review.

A lack of models specific to VCP implementation has lead organizations and researchers to adopting general implementation and evaluation frameworks. More recent VCP implementation studies have adopted the Promoting Action of Research Implementation in Health Services (PARIHS) implementation framework [12,27,35,36]. Kitson et al [36] summarize the PARIHS framework according to 3 key features.

- Evidence: knowledge gathering and engaging leadership, conducting a needs analysis, and identifying initial barriers and facilitators relevant to implementation.
- Context: investigate the quality of the environment or setting where implementation is occurring.
- Facilitation: a strategy that allows interventions to be tailored to enable change and make adoption of a new practice easier.

Implementation success is then defined as a function of these 3 features and the interrelationships among them.

The PARIHS framework has previously been used to implement new services in the public sector. For example, Crowley et al [37] conducted a pilot study to evaluate the feasibility and effectiveness of using existing US VA clinical staffing and equipment to deliver a home-based telemedicine intervention for veterans with diabetes. In their study, the intervention was implemented by members of the US VA (internal facilitators) while the evaluators (external facilitators) managed the research tasks (eg, randomization, outcome analysis) [37]. Other research at the US VA has also employed PARIHS successfully by dividing the facilitation role between internal and external facilitators [27,35].

Although some studies have reported successful implementation using PARIHS, few studies have been found to have used this framework prospectively to design implementation strategies [38]. Furthermore, although one of the greatest benefits of the PARIHS framework is its flexibility and applicability to a variety of different contexts and settings, this can also be seen as a limitation. A review of current evaluation frameworks found that the PARIHS framework was not suited to guiding evaluation, identifying key stakeholders, or generating transferable lessons [38]. An additional limitation of PARIHS is the lack of clarity concerning precisely how the features of the framework facilitate the adoption of interventions on a broader scale (ie, at the organizational level) [39].

### A Proposed Model

From the broader VCP literature and the recommendations made from previous VCP implementation efforts, the following model for the implementation of VCP in public sector health care settings has been formulated. The model has been informed by the latest American Telemedicine Association (ATA) and American Psychological Association (APA) VCP service delivery guidelines [17], as well as the PARIHS and the Shore and Manson [10] implementation models. A detailed overview of the model is provided in Multimedia Appendix 1, which maps the stages and features against previous implementation models, frameworks, and the recommendations of the ATA and APA. Organizations wishing to follow the model can use the VCP Implementation Checklist we developed (see Multimedia Appendix 2).

**Figure 1:**
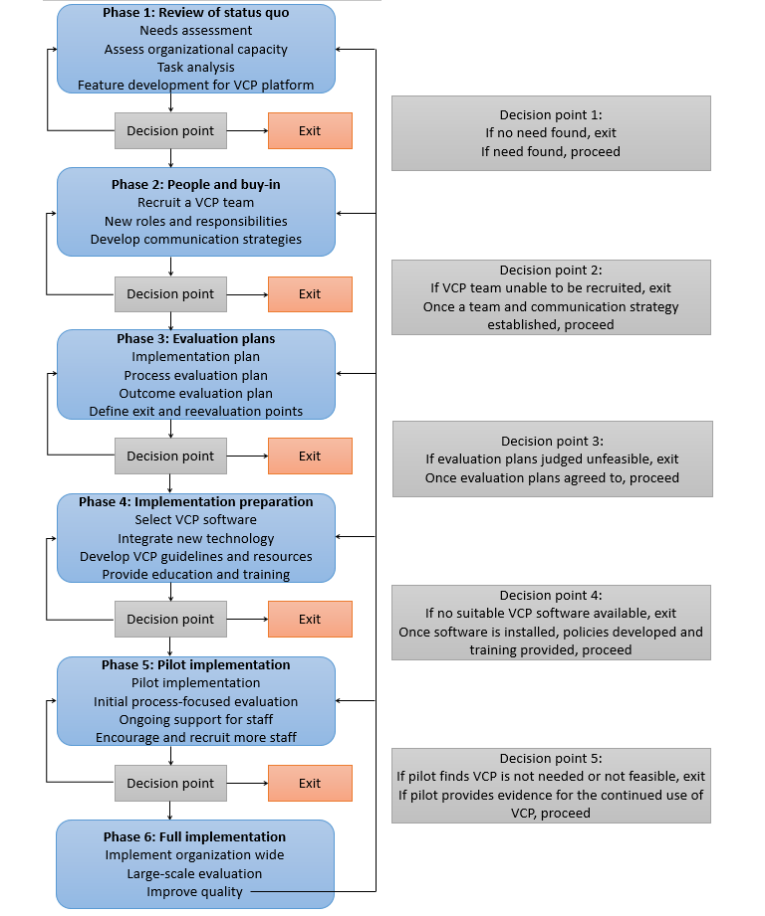
Model for videoconferencing psychotherapy implementation. VCP: videoconferencing psychotherapy.

As shown in Figure 1, the model involves 6 phases: review of status quo, people and buy-in, evaluation plans, implementation preparation, pilot implementation, and full implementation. Working from top to bottom, we describe the major components of the model, and we provide the rationale and relevant literature supporting each step that has been integrated into the model on the basis of the barriers and facilitators encountered in previous research and the models and frameworks used to guide previous implementation initiatives. The model was developed using the cyclical organizational participatory research framework [40]. As indicated by the feedback arrows in Figure 1, the model employs a cyclical and iterative processes that enable organizations to engage with key stakeholders and collect and use data at each stage to help organizations reflect on and integrate findings to improve the process.

### Phase 1: Review of Status Quo

A prominent takeaway from the literature is that before any form of implementation can begin, extensive preparation is required. Doing this work before introducing VCP allows the challenges that have been reported in previous implementation attempts to be identified and addressed. To achieve this, phase 1 has been divided into 4 steps: (1) needs analysis, (2) organizational capacity for change, (3) task analysis, and (4) feature development.

#### 1.1. Needs Assessment

The initial step is to confirm that there is a need for a VCP service and that implementing such a service will fill a gap in the organization’s current service offering. A formal needs analysis can be conducted using a quantitative, qualitative, or mixed approach [41]. As a minimum, the needs assessment should investigate the target population’s experiences and needs, as well as their service delivery preferences and self-efficacy with videoconferencing technology. The organization will then be able to determine whether a VCP service is needed and compatible with the organization’s mission and target population.

#### 1.2. Assess Organizational Capacity

After confirming that there is a need for a VCP service to be implemented, the organization needs to assess its capacity for implementation. Common barriers to the implementation of VCP identified in the literature are technological issues, organizational cultures, clinical workflows, and staffing issues [29]. Therefore, this assessment will be a multifaceted investigation of the technological, environmental, and human resources that are currently available within the organization and what additional resources will be required to implement the VCP service.

Various instruments have been developed and validated to assess an organization’s capacity or readiness to implement telehealth services. A review of telehealth service implementation frameworks [14] highlighted the readiness assessment tool that was developed by Khoja et al [42]. This quantitative measure covers 5 categories of implementation: core readiness, technology readiness, learning readiness, societal readiness, and policy readiness. Organizations should consider adapting this tool to assess their capacity for implementing VCP.

#### 1.2.1. Technological Capacity

Before attempting to implement VCP, the organization will need to assess its technological capacity at the micro and macro-level. At the microlevel, the organization will need to ensure that they have the necessary hardware and mobile technology, as well as sufficient internet capacity. With regard to hardware and mobile technology, the organization will need to ensure that any existing resources (eg, desktops, laptops, and tablets) are equipped with high definition video cameras and audio systems (eg, microphone, external speakers, or headphone accessories). If these features are not built into these devices, they can be added by using external webcams and microphones via USB.

At the macro-level, the organization will need to ensure that their information and communication technology infrastructure is adequate to support a VCP service. Specifically, the organization will need to ensure that the capacity of their internet will be able to provide uninterrupted video communication. A minimum bandwidth of 384 kbps is recommended for conducting VCP [16]. Importantly, the organization will need to ensure that the equipment used to conduct VCP meets the Health Insurance Portability and Accountability Act (HIPAA) requirements, as well as local privacy and data security policies (more information in 4.1 Select Videoconferencing Psychotherapy Software).

#### 1.2.2. Environmental Capacity

In addition to ensuring the organization has the technological infrastructure required to implement VCP, the organization will also need to ensure that adequate space is available to engage in the modality. This may require dedicating a room to VCP. The location chosen to conduct VCP sessions should be well lit and isolated, to limit visual and audio distractions and assure privacy.

The organization will also need to consider the resources available in the community of the target population. To engage in the VCP modality, clients will require the necessary hardware and internet capacity. If the target population is unlikely to possess the hardware or internet capacity (a likely problem for those living in rural and remote locations), alternative arrangements will then need to be considered. The organization will need to determine whether they have the capacity to distribute hardware to clients. If not, organizations may then need to reach out to local community centers (eg, health clinics) that possess the required hardware and capacity (ie, hardware, internet, and room) to enable clients to visit and engage in VCP safely.

#### 1.2.3. Human Factors

With regard to human resources, the organization will need to consider the current capacity of its clinicians to provide VCP. This will involve ensuring that VCP can be introduced so as not to overload clinicians, as well as assessing their readiness to engage in the new modality. Although similar to traditional in-person service delivery, clinicians may not feel prepared to engage in the VCP modality without first receiving some form of formal training. As a result, organizations can likely expect clinicians who have not previously used VCP to have negative attitudes toward the modality.

Previous research has shown that clinicians who have not experienced VCP appear to have more concerns about its effectiveness and the organization’s need for the modality [22,25-27]. For example, clinicians may hold concerns around the effectiveness of the modality, impact on the therapeutic relationship, their familiarity with the technology, dealing with high-risk clients, and concerns with data security issues [43,44]. Therefore, it is recommended that organizations investigate clinician experiences, perceptions of VCP service need, and preferences (or willingness) to engage in a VCP modality.

Clinicians will not be the only ones whose workflow will be disturbed. Clerical and IT workers will also be affected by the introduction of a VCP service. The capacity of the IT support staff to implement, monitor, and provide support for a VCP service will need to be considered. Previous research has found that logistical problems (eg, inadequate staffing, scheduling, overwhelming workloads, and staff changes) are common barriers to VCP implementation [29,45]. Therefore, the introduction of VCP may fundamentally alter the tasks and activities staff perform while delivering in-person services.

#### 1.3. Task Analysis

To assess the human factors and help facilitate later phases in this model, particularly around software selection (4.1 Select Videoconferencing Psychotherapy Software), it may also be useful at this stage to work with clinicians and administrators to generate an in-depth understanding of their daily workflow and key operational tasks in relation to client contact.

A task analysis can be conducted using interviews, focus groups, and structured questionnaires, and it can be documented in writing or diagrams. The primary purpose of these activities will be to generate a features list, identifying the core tasks clinicians and administrators currently perform to deliver services that will need to be supported by the future VCP platform. This involves staff outlining the specific steps they take (both mental and physical) to achieve specific goals [46,47]. For counseling tasks, this could be the following: (1) checking a calendar that is populated by a receptionist for upcoming appointments, (2) accessing a physical client file from a filing cabinet, (3) creating a session plan, (4) going to the waiting room to welcome the client, (5) conducting a session, (6) updating the physical client file, and (7) returning the client file to the filing cabinet. Although this is a broad example, the outcome of this process is a detailed list of activities, currently used tools or software, and relationships among individuals required to complete certain goals. Quantitative measures can also be utilized to complement the task analysis.

#### 1.4. Feature Development for Videoconferencing Psychotherapy Platform

The results from the task analysis can then be used to generate a features list of the core requirements that a VCP platform must have (or can be modified to have), to reduce the friction of adoption at later points. Using the previous example, features for a future VCP platform could include the following: (1) a calendar booking system accessible by clinicians and receptionists, (2) secure Web-based storage for client files, (3) a virtual waiting room for clients, and (4) the ability for the clinician to choose when to start and end a VCP session. A secondary gain of this process is the opportunity to identify pain points for the staff in their current tasks and activities (things that do not work well, are burdensome, or are time consuming) and investigate ways of reducing these pain points through feature selection for the new VCP platform.

The outcomes from this phase will help the organization identify whether the implementation of VCP is feasible and realistic, and these will ensure that the organization is in a better position to determine what protocol and policy changes are needed to facilitate the introduction of VCP. The required content, along with intensity and delivery preferences of training resources, should also be identified at this stage. Finally, addressing relevant human factors at this stage will be helpful to the organization to ensure that any concerns clinical, clerical, and IT staff have are addressed before the time comes to recruit clinicians and communicate the implementation strategy.

### Phase 2: People and Buy-In

As discussed, an organization might expect some resistance to VCP implementation from clinicians. If drastic changes from previous protocols are required, the changes may also result in resistance from clerical and IT staff. Therefore, it will be important that the organization manages this change effectively and is able to foster buy-in for the VCP modality.

The Diffusion of Innovation theory is a valuable change model for guiding technological implementation initiatives. This theory appears particularly relevant to VCP, as it highlights the importance of communication and peer networking while accepting that not all organization members will adopt the new modality immediately [48]. The theory posits that an initial few people will be open to the new technology and adopt its use before more people become willing to try and eventually adopt the technology. This phase has been broken down into 3 steps in an attempt to apply this theory: (1) recruit a VCP team, (2) new roles and responsibilities, and (3) develop communication strategies.

#### 2.1. Recruit a Videoconferencing Psychotherapy Team

Implementation initiatives in public sector health care organizations are often at risk because of their complicated bureaucracies. Given the multifaceted and cross-collaborative nature of VCP, coordination could prove to be particularly difficult and time consuming, putting the implementation of the service at risk [30]. To overcome this challenge, a critical step for implementing VCP is the recruitment of a motivated and stable (ie, permanent) team to lead the implementation. These individuals can be viewed as change agents, and they will predominantly be responsible for identifying and engaging with key stakeholders and developing a positive VCP culture [49].

Given the cross-departmental collaboration required to implement VCP, it will be important to ensure that each department within the organization is represented on the team. The composition of this team should therefore include organizational leaders, clinicians specializing in the offering of VCP, on-site VCP champions who encourage and promote VCP to clients and clinicians, IT support staff, and clerical staff representatives. Involving representatives from each department will help ensure that all parts of the organization have a voice in the implementation process. External representatives may also be recruited as consultants if an organizational need for this is identified in Step 1.2 (Assess Organizational Capacity).

It is important for these team members to be dedicated and committed to the implementation process. This team will need to appoint a leader who is ultimately responsible for coordinating the implementation. This person should have the authority to execute any decisions made across the organization. Given the time-intensive nature of implementation, the person assigned this role may need to make the management of the implementation his or her full-time role.

#### 2.2. New Roles and Responsibilities (and Personnel)

The introduction of VCP will likely bring about changes to the roles of current clinical, clerical, and IT staff [50]. The organization will need to be transparent and work closely with existing staff to effectively manage these changes.

#### 2.2.1. Videoconferencing Psychotherapy Champions

Program champions have long been recognized as critical resources for implementation [51]. Previous VCP implementation studies have found champions to be vital facilitators [10,12,22,25,27,52]. VCP champions will play a key role in providing ongoing support and information to participating personnel, acting as a communication bridge between the staff on site and those in key management positions, relaying concerns and directions accordingly. Champions may take on additional responsibilities, including potentially hosting VCP training and personal development seminars for clinicians.

The champions will need to foster a sense of enthusiasm toward VCP at the clinic level. This may be a challenge, given some clinicians may be skeptical of the modality. But if successful, the champion will lend support to the credibility, trial, and eventual adoption of VCP. To be successful, champions should exhibit homophily (ie, high similarity with other staff), empathy, and openness [49]. For large organizations with multiple, geographically dispersed service providers, it is recommended that each provider nominate its own VCP champion.

As staff concerns may center around VCP use in practice, when feasible, it may be useful for champions to gain some experience in conducting VCP themselves, before the initiation of the implementation, so that they can speak to their colleagues from experience. Once the VCP software is installed, champions may consider using the software to perform everyday tasks (eg, conduct meetings with other staff). Using the software in this way, in a low-risk environment, may be beneficial for increasing staff confidence with the technology.

#### 2.2.2. Telehealth Coordinator

Introducing VCP may also create opportunities for new roles within the organization. An example of such a role is a telehealth (or VCP) coordinator. This person will be responsible for managing the VCP technology and process (eg, alleviating technical difficulties and managing appointments and consent forms).

It is recommended that organizations employ a technician dedicated to VCP, as previous studies have found that such roles help facilitate implementation [27,52]. This role may be filled by an existing staff member who possesses the knowledge and passion for the VCP modality. Alternatively, the organization may need to advertise to fill such a role.

#### 2.3. Develop Communication Strategies

Communication channels will be needed so that concerns and solutions to any difficulties encountered during the implementation of VCP can be communicated effectively. Communication deficiencies have been identified as a key barrier in previous implementation studies [53]. This may be particularly true for VCP implementation efforts, as implementation will involve a variety of individuals from different departments, including clinical, clerical, and IT staff, as well as center management and organizational leaders. Therefore, the fostering and development of communication channels among these stakeholders, as well as clients, is another important step.

The first important point of communication will be to apprise clinicians and clients of the need for VCP. Communicating this need will require a strategy that is consistent over time and geographical region. Visits to participating clinical sites and regular teleconference calls with clinicians and meetings with clinical advisory committees have been reported to be helpful for ensuring successful implementation [10,35]. Messages related to the progress of VCP implementation should also be a standard agenda item at clinical meetings and appear in newsletters or bulletins prepared for clinicians and clients. Additional communication strategies for promoting VCP to clients include promotional materials and expressions of interest via the organization’s social media channels, webpages, and newsletters, as well as signage and materials (eg, brochures) within facilities and presentations at local community events.

### Phase 3: Evaluation Plans

Timelines reported in implementation studies to date suggest that it may take between 1 and 12 months for activities in phases 1 to 4 to be completed [10,37]. Therefore, extensive preparation and forward planning are essential for the success of VCP implementation. This will require the organization to develop an implementation and evaluation plan by using a logic model, with goals and objectives for the new service, and a set of key variables (or success indicators) to be used to evaluate the implementation and effectiveness of the VCP service. Organizations should use this plan to assign the timeframe for each activity, as well as the specific personnel responsible for each activity. To enhance buy-in, all stakeholders should be involved in the development and approval of this plan.

#### 3.1. Implementation Plan

The implementation plan should contain a project timeline, which lists the processes and milestones required to (1) install the VCP technology (see 4.2 Integrate New Technology) and (2) collect data for an evaluation (see 3.2 Process Evaluation Plan) and explain how the activities will be delivered within the stipulated timeframes. It is recommended that organizations employ a staged implementation approach. VCP should initially begin at a single site before being introduced more extensively throughout the organization’s other service providers. This approach has previously been found to be effective for implementing VCP services in public sector organizations [10,27,52], and this approach has several advantages over simultaneous implementation approaches. For example, a staged approach starting at a single location affords the organization the opportunity to gain meaningful insights into implementation processes that can later be applied on a larger scale. Such an approach also ensures that implementation can be tailored to accommodate the unique characteristics and available resources for each site. This planning phase will also help foster confidence in the efficacy of VCP, and, if seen as successful, this may promote the adoption of VCP by more clinicians and clients.

There are several factors that an organization should take into consideration when selecting a site for pilot implementation. These include the human, organizational, and community factors identified in Step 1.2 (Assess Organizational Capacity). The selected center should have the greatest readiness for VCP implementation. This means that the selected site must have personnel (ie, clinicians, clerical, and IT staff) who are available and willing to implement VCP. For example, the readiness of clinicians, in terms of experience or openness to adopting new technology needs consideration. Selecting a site with clinicians who are more amenable to VCP and more experienced in its use will assist the initial implementation of the service. The selected site will also need to have the necessary resources to deliver VCP (eg, computer hardware and space). The local client demand for VCP should also be considered. Demand may be higher for sites with a higher level of clinical need or where clients may find particular value in therapy via a Web-based modality (eg, in sites serving individuals in rural or remote locations).

#### 3.2. Process Evaluation Plan

Evaluation forms a key part of monitoring implementation and determining its impact. Evaluation can be divided broadly into 2 activities, which might be a focus during different stages of implementation: process evaluation (investigating the successfulness of the implementation) and outcome evaluation (investigating the impact of the VCP service).

The evaluation plan needs to be considered at an early stage so that appropriate baseline measurements can be incorporated into service delivery. The process evaluation will encompass collecting data from those involved in the implementation to determine how the intervention was actually implemented. Data collected should aim to answer questions regarding the success of the implementation; the following are examples of such questions: Has VCP been implemented as intended? Were there any unanticipated barriers to implementation? How could implementation be improved? Are additional resources required for clients, clinicians, or administrative staff? Is it feasible to expand the service to other sites?

#### 3.3. Outcome Evaluation Plan

On the contrary, when considering the outcomes of implementation, questions may include the following: Has availability of VCP increased delivery of therapy? Are treatment needs better met in particular groups that were hoped to benefit from greater accessibility of VCP (eg, persons in rural or remote areas)? Are clients satisfied with VCP? Is overall satisfaction with the service improved in people who have accessed VCP? Does receipt of VCP result in improved clinical outcomes?

Many of these questions can be answered by comparing the outcomes for a group of VCP clients to control groups. However, whether and how to use control groups for VCP evaluations require some careful consideration in relation to constraints of how feasible comparisons will be when considering anticipated numbers, sources of error and bias, and the statistical reliability of measurement.

#### 3.3.1. Outcome Evaluation Data Considerations

Evaluation implicitly involves comparison, which may be defined in terms of change over time (eg, is delivery improved on some metric relative to previous indicators?) or between parallel versions of service delivery (eg, does the addition of VCP improve individual outcomes compared with routine service delivery?). Collecting these data may require planning to administer a measure during a baseline period before VCP implementation for comparison or to a group that is not receiving VCP to provide comparison data for the evaluation. Metrics, such as receipt of VCP or in-person therapy, may be relatively easily captured, but satisfaction and clinical outcomes may require incorporating new measures into practice.

Measures of uptake or delivery of therapy should be simple enough to collect routinely and accurately. If VCP supplements in-person therapy, pre-post implementation comparisons of total therapy delivery are likely to be meaningful. Such comparisons might be strengthened by obtaining data from a site not yet offering VCP to control for the effects of other organizational or external changes that may arise during the implementation period (eg, change in management or referral pathways).

However, achieving sensitive measurement of client clinical outcomes may be more challenging. Although clinical outcome measures may already be routinely administered, there may be significant missing data, resulting in systematic bias if there is a lack of follow-up data on clients discontinuing therapy. Client and intervention heterogeneity may mean that broad catchall measures of clinical outcome or quality of life need to be used. Universal measures, such as general well-being, overall symptom measures (eg, the Depression Anxiety Stress Scale; the Brief Symptom Inventory), or mental health–related quality of life (eg, The World Health Organization Quality of Life, Assessment of Quality of Life-8D) may be suitable for capturing outcomes across a number of client groups. However, it should be noted that the use of broad measures can mean reduced sensitivity to individual outcomes. Considerations in providing more sensitive measures include reviewing the most tailored measures, focusing evaluation on a specific subgroup where more sensitive measures can be used, resourcing personnel to ensure measures are administered, and follow-up with clients who discontinue therapy. A comparison group of clients not receiving VCP at another site might be used to control for the natural course of improvement under treatment as usual. However, given that effect sizes for many in-person psychotherapies are in the small-to-moderate range under trial conditions [54], in practice, it may be difficult to differentiate effects of VCP from site differences and other sources of error. Therefore, there may be value in adopting a randomized controlled design during a stage of the implementation, comparing with a treatment-as-usual or waiting-list group to control for the natural course of improvement.

#### 3.3.2. Outcome Evaluation Design Considerations

Alternative designs, such as quasi-experimental designs, may be considered. Although these methods are not without their limitations, such as selection bias, they may provide valuable insight into differences between the effectiveness of VCP and in-person therapy. Other possible designs include the regression-discontinuity design and interrupted time-series design [55]. Psychological interventions typically produce small incremental changes in outcome measures, which may require several hundred participants to make meaningful between-group comparisons. Therefore, consideration to whether it is realistic to obtain sufficient data for comparisons to be meaningfully made is required.

In services that are introducing VCP as an alternative to in-person therapy, although it may be tempting to compare modalities to determine whether equivalent outcomes are produced, it should be noted that demonstrating noninferiority of an intervention requires higher statistical power than a superiority comparison [56]. As any differences on the basis of mode of delivery are likely to be very small, they are likely to be obscured by error and bias, unless a highly rigorous randomized controlled trial design were possible with a very large sample. In making comparisons between VCP and in-person therapies, it may be more realistic to compare metrics more proximal to the process of therapy, such as satisfaction and working alliance ratings.

#### 3.4. Define Exit and Reevaluation Points

It is also important to consider the steps to be taken following data analysis. Results should, where possible, feed into the decision-making process when moving between steps in this model (Figure 1). There may be a number of critical decision points for each organization at which the results obtained through evaluation suggest the next steps to be taken. Articulating these steps and integrating them into the implementation and evaluation plan may save larger organizations time and resources at later points by clearly identifying the thresholds at which the implementation (1) proceeds to the next step, (2) regresses to an earlier step to reevaluate and adapt from the learnings of the most recent evaluation (eg, consider a different software selection), or (3) exit if the implementation does not appear to be feasible in the current organizational environment. These decision points allow the model to be flexible to changing needs and explicitly introduce feedback loops that may be useful for the ongoing quality assurance of the software system and service provision.

### Phase 4: Implementation Preparation

This phase involves organizing all the appropriate resources for VCP implementation. Any gaps between the current and required resources identified from the assessment of organizational capacity should be acquired before any implementation attempt.

#### 4.1. Select Videoconferencing Psychotherapy Software

The most critical resource to acquire will be the software platform to facilitate the VCP service. This choice is not simply a matter of cost. To aid in decision making, a features list (generated in Step 1.4 Feature Development for Videoconferencing Psychotherapy Platform) can be used to map existing software platforms against the core requirements and nice-to-have features of the organization (ie, clinician and clerical staff wish lists). This features list should have input from all relevant stakeholders and may require some specialist input (eg, lawyers to advise on appropriate terms of use).

This highlights the need for organizations to reach out to potential software providers to determine which one will provide the best fit. Organizations may wish to consider creating a shortlist of potential providers and making contact with each to discuss items regarding system integration (eg, customer support, access to resources, and customization) to help determine the best choice. The organization will also need to consider the security of each platform and the user experience each platform offers.

#### 4.1.1. Data Security

Data security and bandwidth should be kept at the forefront of the decision making when selecting a suitable platform. Data security is a particular concern in any health care setting, as it is the organization’s responsibility to ensure that client health data are kept confidential. Similar to any Web-based service, VCP brings a range of data security risks with regard to computer-mediated communication. For clients, concerns may revolve around geolocation vulnerability and hacking. However, these issues are likely only if one’s computer is compromised rather than the actual software and having an up-to-date antivirus program should alleviate these concerns.

A more significant concern for organizations revolves around data encryption and the transference of data involving third parties. The organization should ensure that the technology meets relevant privacy requirements, such as HIPAA standards for protecting health information that is held or transferred in electronic form. Currently, there are a number of HIPAA-compliant platforms available to organizations for conducting VCP, including the following: VSee, Cisco (Cisco Systems, Inc), Polycom (Plantronics, Inc), and CoViu. Skype for Business (Microsoft Corporation) is also HIPAA-compliant, as is Zoom (provided the organization signs a Business Associate Agreement before use). Organizations are encouraged to consult their own IT experts to investigate the different VCP platforms before deciding which would be the best fit for the organization.

It will be the organization’s responsibility to communicate risks to clients and have measures in place to mitigate the security risks outlined above. To overcome technology obstacles, it is recommended that binding contracts are put in place for ongoing support and continuity of service, data security, and technical support. Such contracts would protect against severed services and ensure that technological support is available to the organization when needed.

#### 4.1.2. Usability

Another critical and often overlooked consideration is the software’s usability. End users for VCP can include clients, clinicians, administrators, and IT support. It is possible for a software platform to meet organizational and legislative data security requirements, but it is also possible for it to be too challenging to learn, use, and adapt to the daily tasks and processes of staff. Trialing a variety of software platforms in low-risk environments (eg, to conduct remote team meetings) may help to highlight any usability issues. Modifications to the software, a choice of different software, or changes to team processes may need to be considered at this point to find the best fit between the usability of a system and the technical feature requirements of a platform.

Some key questions may be asked at this stage, which can form a part of usability testing: What did you like about software platform A compared to platform B? How long did it take you to book an appointment in software platform A compared to software platform B? How confident were you in using the platform? What did you find most difficult? What resources would you need to make using the platform easier? A cognitive walkthrough may also be useful for identifying the tasks, challenges, and nontypical behaviors occurring on a system that may act as barriers to or facilitators of ease of use [57-59].

At an organizational level, branding may also be an important consideration. If this is a relevant consideration, selecting white-label software (ie, purchasing a platform or license produced by a particular company but rebranded by the organization to make it appear to be their own platform) may be a priority. Alternatively, the organization may decide to embark on a software development process.

#### 4.2. Integrate New Technology

The organization should not underestimate the time taken between software implementation and uptake by clinicians. Simply installing the software on clinicians’ computers will not mean that clinicians will automatically be able to use it. Therefore, once the organization has selected a software provider, careful planning, communication, and training will be required to help expose clinicians to the platform and build confidence in its use. Once the technology has been selected, the organization will need to come up with a process to integrate this platform into its existing procedures. This may mean that the organization’s current appointment scheduling, client monitoring, consent form distribution and collection, and client data or medical records systems need to be altered to accommodate the VCP platform and process. The process of integration will likely vary widely on the basis of an organization’s current systems and the functions of the newly selected VCP software; therefore, an in-depth discussion of technology integration is not offered here. For an in-depth discussion of technology integration, organizations are recommended to refer to Pfeiffer [50].

#### 4.3. Develop Videoconferencing Psychotherapy Guidelines

The introduction of VCP will likely require new policies or amendments to existing policies and procedures within the organization. This will require careful planning. In addition to establishing any changes to workflow, training requirements, and responsibilities of existing (or new) staff, the organization will need to consider policies involving informed consent for VCP and crisis management procedures. We encourage organizations to follow the recommendations put forth by Luxton et al [60]. Shore et al [17] outline several areas that will need to be considered when developing policy and operating procedures, such as roles, responsibilities, communication avenues and emergency procedures, agreements for training, and evaluation processes.

#### 4.4. Develop Other Resources

Other resources, such as training materials and facilities (eg, clinical rooms), are essential for VCP. Once procured, all equipment and procedures should be rigorously tested to ensure that the platform works successfully before any implementation attempt. The workflow should also be tested to ensure that the entire system (from clerical staff scheduling an appointment to the clinician concluding a session) is working.

Organizing resources for clients should be another consideration in any implementation strategy. Access to all the required technology for the clients, including a good internet connection, is needed for VCP. This may be a critical issue because of the geographical dispersion of clients in the case of large organizations, which may affect the quality of internet services and the quality of the VCP. Organizations should consider having local rural sites, for example, a room in a medical center, which are accessible and equipped with all the required software and facilities for a client to engage with VCP. Such an arrangement would also address safety concerns for at-risk clients.

Reducing the burden on the client in terms of cost, and learning how to use the VCP technology, may encourage more individuals to take up this service, and this should be an important consideration in any VCP implementation attempt. Therefore, it is recommended that organizations have an employee available to clients to help facilitate the initial VCP appointment, perhaps also introducing the client to the VCP platform. In addition, the organization may consider developing instructional resources (eg, video demonstrations and PDF manuals) to assist clients setting up and preparing for VCP.

#### 4.5. Provide Education and Training

Although VCP attempts to emulate the in-person experience as closely as possible, clinical staff will need to develop new skills that are required to successfully engage in VCP. Therefore, the organization must offer training and support to the clinicians around the provision of VCP services.

First, clinicians will need to be trained on how to navigate the technology. This training will provide an opportunity for the clinicians to become familiar with the VCP technology, procedures, and logistics of using the new modality. In particular, special VCP processes may be needed for booking appointments, distributing handouts, establishing client rapport, monitoring client comfort during sessions, and troubleshooting. These processes have been reported as barriers to delivering VCP in previous research [61]. Experiential learning (eg, role play) may be particularly useful here.

Training around supporting high-risk clients is also very important. Inadequate training of clinicians was identified as a key barrier in previous implementation strategies [22,25]. Even in in-person therapy, some clinicians have reported concerns around using trauma-focused treatments, because of fears of retraumatization and increased dropout [62]. Therefore, it is understandable that clinicians may hold reservations about using such approaches with VCP, despite studies demonstrating VCP to be safe and effective when using interventions, such as cognitive behavioral therapy and Prolonged Exposure for posttraumatic stress disorder populations [63,64]. However, Tuerk et al [63] did note that clients with more severe symptoms, such as high levels of hypervigilance, may be more difficult to treat with VCP. Training and identification of safe and effective approaches that can be used with VCP for such clients is therefore of paramount importance. Organizations may consider excluding clients entirely from receiving VCP when cognitive capacity, previous history of violence, or self-harm suggest that this is necessary. This is particularly necessary when geographic distance to the nearest medical facility and the lack of a local support system are of concern [17].

The goal of this training and education should be to alleviate several concerns clinicians hold around the effectiveness of VCP and any safety issues, allowing them to develop skills for working with complex mental illnesses, such as posttraumatic stress disorder, including risk management skills. The education and support of key clinical staff should be ongoing. This will lead to an increase in confidence and ultimately an increase in uptake. Organizations are encouraged to consult recent training and education guidelines from the ATA [17,18].

Delivery of this training could be a combination of both Web-based and in-person learning. A combination may facilitate buy-in and, at the same time, reduce cost and required resources. Organizations may consider developing a Web-based resource dedicated to VCP. Such a resource would provide staff with a centralized portal to access core and supplementary training materials and support (eg, a discussion board to engage with peers), as well as other relevant resources (eg, quick reference VCP pdf manuals). Having an accessible, single point of contact for follow-up support (eg, a telehealth coordinator or VCP champion) is also advised.

Ultimately, the organization should aim to keep training as comprehensive but as brief as possible. As discussed earlier, there may be some resistance to implementation from clinicians because of workloads. Clinicians will not want to spend any more time than is absolutely necessary to become proficient in using VCP. Organizations should therefore aim to keep training brief and to the point.

Finally, training will not be limited to clinicians. Clerical staff will also need to be trained in how to execute any new or modified tasks that have resulted from the introduction of the VCP service offering (eg, scheduling appointments, emailing instructional resources, and obtaining consent from clients).

### Phase 5: Pilot Implementation

Once all the groundwork has been completed, establishing a sufficient base and framework for VCP implementation, phase 4 may commence. This phase has been broken up into the following 5 steps: pilot site implementation, process evaluation, provide ongoing training and support, recruit and engage more clinicians and clients, and demonstrate meaningful use. As with any pilot evaluation, these steps are likely to take between 6 and 12 months (depending on organizational capacity). As demonstrated in Figure 1 and explained below, the pilot phase also provides a critical opportunity to exit or reevaluate the implementation approach before significant time and finances are invested.

#### 5.1. Pilot Site Implementation

It is recommended that the implementation process commence at 1 site [10,50]. This will be advantageous in identifying barriers and developing strategies to help overcome difficulties as they arise. Informed by the first 4 phases in this model, the development of an effective and uncomplicated strategy for the initial site will facilitate VCP implementation organization wide. For increased control, the organization may consider restricting this pilot to a limited number of clinicians and clients and for a fixed period of time [50]. In addition, the type of therapy and client might also be restricted during this initial pilot phase to those that are best suited to VCP. For example, managing high-risk clients via VCP may be a daunting prospect for clinicians. By removing such clients from the initial pilot, clinicians can focus on becoming accustomed to the modality.

In a further effort to ensure the pilot is a success, it will be important to ensure that participation is voluntary. Only clinicians and clients who want to engage in VCP should participate in the initial pilot. This should improve the likelihood of success, as the clinicians and clients will have a vested interest in making things work (and will be more persistent if barriers are encountered).

Once VCP has been successfully implemented at the pilot site, implementation can then occur at additional sites (one at a time).

#### 5.2. Initial Process-Focused Evaluation

To ensure that the implementation plan is optimized, an evaluation should be simultaneously conducted on the basis of the plan developed in Step 3.1 (Implementation Plan). This initial evaluation will likely focus on process evaluation and potentially some preliminary examination of outcomes. With a small number of clients seen at this stage, it may not be possible to collect meaningful outcome data. However, quantitative data can be captured on feasibility and acceptability, using indices such as client uptake, numbers of sessions attended per client, nonattendance rates, dropout, out-of-session time spent per client, and client ratings of satisfaction. Alongside this, it will be useful to set up recording procedures for capturing data on the frequency of technology problems encountered, as well as any adverse events. Data collection and storage methods should be tested before implementation.

Qualitative data (via interviews) should also be collected at this time, Interian et al [22] provide an example interview schedule. The feedback collected from key stakeholders (eg, clients, clinicians, leaders, clerical, and IT staff) will assist in identifying challenges and barriers to VCP implementation. It is likely that those interviewed may offer solutions to overcome any difficulties experienced in the future.

Taken together, data collected for the process evaluation will allow the organization to identify strengths and weaknesses in the implementation plan. The results should inform any modifications required to the implementation plan and any decisions to modify or acquire additional resources, training materials, and policies. These decisions may already be defined (Step 3.4 Define Exit and Reevaluation Points). The results of the process evaluation and any changes made should then be communicated to all key stakeholders. Organizations may consider conducting seminars directed to its service providers, summarizing the initial implementation effort and the lessons learned.

It is likely that the pilot site implementation will cycle through several brief single-site iterations to integrate the findings of the process evaluation. As outlined in Step 3.4 (Define Exit and Reevaluation Points), the decision threshold for moving forward from a single-site pilot should be defined as a part of the implementation and evaluation plan, as well as thresholds for reevaluating and returning to earlier steps.

#### 5.3. Provide Ongoing Support and Training for Clinicians and Staff

As identified in earlier stages, training and support for clinicians, as well as the wider team, is fundamental in overcoming implementation barriers and therefore critical to any implementation attempt. This support and training should be ongoing, addressing concerns as they arise and facilitated by the on-site champion. Clinicians will have needs in staying up to date with evolving VCP technology, research findings, and policies [17], which may need to be factored into training and clinician workload allocations.

At this time, the organization may wish to consider launching a mentoring program to help promote VCP, recruit clinicians and clients, and assist with the sharing of best practices. This has been found to be helpful for establishing newly implemented service offerings in geographically dispersed organizations [65].

#### 5.4. Encourage and Recruit More Clinicians and Clients

Communication about the VCP implementation is critical for confirming the need for VCP and for disseminating best practices for VCP. This communication should be directed to clients and clinicians, with a view to encouraging additional clinicians to obtain VCP training. Again, this step will likely involve local champions promoting VCP.

Drawing on a greater pool of experiences and knowledge will help identify barriers and methods to overcome problems. The local champion may need to organize information and training sessions to facilitate recruitment of clinicians or promotional flyers to help recruit more clients.

#### 5.5 Demonstrate Meaningful Use

As explained in Step 3.2 (Process Evaluation Plan), conducting a process-focused evaluation may introduce variables to service delivery that would ultimately not form a part of daily practice. With this consideration in mind, a period of meaningful use at the pilot site may be beneficial. This provides both staff and clients with the opportunity to engage with the VCP system, as it is intended to be used at scale.

### Phase 6: Full Implementation

The next step is to implement the VCP service organization wide. This final phase involves 3 further steps; implement VCP organization wide, ongoing process evaluation, and, finally, outcomes evaluation.

#### 6.1. Implement Organization Wide

Once phases 1 to 5 have been successfully completed, implementation of VCP on a wider scale may be considered. It is presumed that several challenges will have been addressed and a successful model of VCP established to help with this transition. It is recommended that the rollout should be a slow process to ensure participating sites are not rushed into the introduction of VCP and ensure they have sufficient time to organize their staff, training, and resources.

Education at site level should be thorough, and ongoing support should be available to clinicians from software suppliers and IT staff. Site-based VCP teams will need to be organized, and a local champion at each site is required to help facilitate communication, ongoing training, and problem solving. Organizations may also consider relocating willing staff from the initial pilot site to the next site, temporarily, to assist with implementation.

#### 6.2. Large-Scale Evaluation

Using the plans developed in phase 3, the organization can now set out to evaluate the implementation and impact of VCP. The process evaluation should involve feedback from the key stakeholders, such as the clients, clinicians, and managerial staff to ensure their needs are being met and that a high standard of service delivery is maintained. This evaluation will also address any remaining problems or deficiencies.

Once organization-wide implementation is established, there may be sufficient data to examine the impact of VCP. Ideally, processes for data collection should integrate seamlessly into current clinical practice; however, the additional work involved with collecting consent, measures, and questionnaires, as well as collection of feedback from their clients to provide data for the outcomes evaluation, may make clinicians hesitant to participate in an evaluation. Methods for reducing this workload therefore need to be explored. Electronic consent processes may be helpful [17,66]. Having a dedicated external evaluation team with appropriate skills is an important consideration for an outcome evaluation. Employing an external team to perform the evaluation can help the organization to focus on service delivery, and this is especially useful for those organizations that may not have the capacity to conduct rigorous evaluations. Having an external evaluation team that is independent of the delivery of VCP has been found to be effective [27,37]. The team should be identified early in the implementation initiative to help develop the implementation and evaluation plan that is discussed in phase 3. This will ensure that those responsible for the evaluation can collect the necessary data to properly evaluate the service. Organizations are advised to refer to established guidelines for commissioning and executing evaluations [67-69].

#### 6.3 Improve Quality

Once implemented, the steps in this model can be revisited to conduct targeted quality assessment and evaluation. For example, many software products may come to the end of their lifecycle as operating systems, and consumer preferences and organizational needs change. Returning to phases 1 to 4 may assist organizations in the transition to new technologies, with limited disruption to the workforce. Alternatively, regular scheduling of evaluation activities in phases 3 to 6 may assist with ongoing quality control. Therefore, the model presented here is not only applicable to initial implementation but can also be used postimplementation to assist organizations to modify or transition components of their established VCP service.

## Discussion

### Principal Findings

We have developed and described an integrative implementation model for VCP in large organizational settings in the public sector. A strength of this model is how it draws on multiple fields of research across mental health, IT, and organizational psychology to overcome the limitations of previous models and frameworks used in VCP implementation projects (see Multimedia Appendix 1). Organizations and researchers looking to implement VCP via this model are encouraged to use the VCP Implementation Checklist (see Multimedia Appendix 2).

The search strategy used to identify papers in our review of the VCP literature was not systematic, meaning our search was potentially biased and the quality of studies cited left unassessed; this is a limitation of our paper. Future research should consider a more systematic review of the literature to provide a more detailed assessment of the barriers, facilitators, and lessons learned from VCP implementation studies. In addition, this model highlights the need for additional research to be done regarding implementation of VCP into large organizations that do not target veterans. The model developed in this paper draws heavily on the experience of implementing VCP in the VA. Although this represents a large and geographically disperse organization, some of the challenges may be specific to veteran and military contexts and therefore may not translate to all organizational environments. It is therefore expected that this model will continue to evolve as researchers and organizations apply the model and report evaluation findings in nonmilitary environments.

### Conclusions

The time and resources required to successfully implement VCP in large organizational settings, which are often geographically dispersed, are likely to be extensive and should not be underestimated. It is expected that the entire process may take several years to successfully complete, but the provision of mental health support via the internet has a critical role to play in reaching vulnerable individuals with limited access to such care. The ability of this modality to overcome barriers faced by clients cannot be ignored. It is hoped that the prescribed model will be useful for researchers and organizations to help guide and optimize VCP implementation efforts in the public sector in the future.

## Appendix

Multimedia Appendix 1 Staged approach for videoconferencing psychotherapy implementation.

Multimedia Appendix 2 Videoconferencing psychotherapy implementation checklist.

## Abbreviations

APA: American Psychological Association
ATA: American Telemedicine Association
HIPAA: Health Insurance Portability and Accountability Act
IT: information technology
PARIHS: Promoting Action of Research Implementation in Health Services
VA: Veterans’ Affairs
VCP: videoconferencing psychotherapy
