# Supplementary material for: Videoconferencing Psychotherapy in the Public Sector: Synthesis and Model for Implementation
Source: JMIR Ment Health. 2020 Jan 21;7(1):e14996. doi: 10.2196/14996 (PMC7001045; doi:10.2196/14996)
Supplement: Multimedia Appendix 1 [file mental_v7i1e14996_app1.docx]

| **Stage** | **Summary** | **PARIHS** | **ATA & APA [17]** | **Shore & Manson [10]** |
| --- | --- | --- | --- | --- |
| **Phase One: Review of Status Quo** |  |  |  |  |
| 1.1. Needs assessment | - Establish justification for the service implementation  - Identify the needs and preferences of clients | X | X | X |
| 1.2. Assess organisational capacity | - Inventory of currently available human, organisational and community resources relevant to VCP implementation and service delivery | X | X | X |
| 1.3. Task analysis | - Generate an understanding of the specific processes in staff roles | X |  |  |
| 1.4. Feature development for VCP platform | - Generate a features list for VCP platform |  |  |  |
| **Phase Two: People and buy-in** |  |  |  |  |
| 2.1. Recruit a VCP team | - Recruit a team of motivated and permanent staff with representatives from each department within the organisation. |  |  |  |
| 2.2. New roles, responsibilities (and personnel) | - Recruit VCP champions at each site.  - Hire a VCP coordinator |  |  |  |
| 2.3. Develop communication strategies | - Develop a strategy to inform staff of the need for VCP. Develop a plan that involves consistent messaging and regular communication between stakeholders. | X |  |  |
| **Phase Three: Implementation preparation** |  |  |  |  |
| 3.1. Design implementation and evaluation plan | - Produce a logic model with goals and objectives for VCP.  - Establish a set of key variables (success indicators) to be used to evaluate the implementation and effectiveness of the VCP service. | X |  |  |
| 3.2 Define exit and re-evaluation points | - Identify critical decision points in the implementation model  - Define actions to be taken in response to evaluation results. |  |  |  |
| 3.3. Develop guidelines | - Develop new and/or amend existing policies and procedures to accommodate new VCP service. | X | X | X |
| 3.4. Procure resources | - Procure a VCP software platform and other resources identified as necessary and lacking from the assessment of organisational capacity. | X | X |  |
| 3.5. Provide education and training | - Educate clinical staff on how to use the technology as well as how to engage with specific populations. | X |  |  |
| **Phase Four: Pilot implementation** |  |  |  |  |
| 4.1. Pilot site implementation | - Select a single site to implement VCP  - Implement VCP at this site | X |  | X |
| 4.2. Initial process-focused evaluation | - Collect quantitative data measuring feasibility and acceptability of the VCP service.  - Collect qualitative data from key stakeholders discussing challenges and barriers to VCP implementation.  - Analyse data to identify strengths and weaknesses of the implementation strategy.  - Make any modifications that are necessary prior to further implementation attempts.  - Communicate results to key stakeholders. | X |  | X |
| 4.3. Provide ongoing support and training for clinicians and staff | - Provide ongoing training to clinicians  - Consider developing an online centralised resource for staff to access support. | X |  |  |
| 4.4. Encourage and recruit more clinicians and clients | - Promote the use of VCP. | X |  |  |
| 4.5. Meaningful use | - Promote a period of meaningful use of the VCP platform outside of a pilot study context.  - Examine VCP processes as they would occur “in-the-wild” |  |  |  |
| **Phase Five:**  **Full implementation** |  |  |  |  |
| 5.1. Implement organisation wide | - Implement VCP at remaining sites. | X |  | X |
| 5.2. Large scale evaluation | - Complete evaluation of VCP service. | X |  |  |
| 5.3. Improve quality | - Re-visit earlier phases of the model to ensure resources and the service is of the highest quality. |  | X |  |
